# 519. DeepLPI: A Novel Drug Repurposing Model based on Ligand-Protein Interaction Using Deep Learning

**DOI:** 10.1093/ofid/ofac492.574

**Published:** 2022-12-15

**Authors:** Bomin Wei, Yue Zhang, Xiang Gong

**Affiliations:** Princeton International School of Mathematics and Science, Princeton, New Jersey; School of Medicine, the University of Utah, Salt Lake City, Utah; Princeton International School of Mathematics and Science, Princeton, New Jersey

## Abstract

**Background:**

Drug repurposing has gained increased attention because it proposes to find effective cures for new diseases from approved drugs to lower development costs and time, and computational prediction of protein-ligand interactions (PLI) can provide accurate and fast drug screening. The shortcomings of existing machine learning-based methods for PLI prediction include 1) using human-selected features leads to loss of information and therefore lower accuracy and 2) using limited 3D structure data for input leads to lower generalizability.

**Methods:**

To address the shortcomings, I proposed DeepLPI, a novel deep learning-based model that takes as input the raw sequences of drug molecules and proteins. DeepLPI applied pre-trained embedding models to encode the raw sequences into dense vector representations, which were then fed into 1D-CNN and biLSTM to obtain predictions. BindingDB dataset was used for model training and performance evaluation that is compared with a start-of-the-art method DeepCDA.

**Fig 1.**
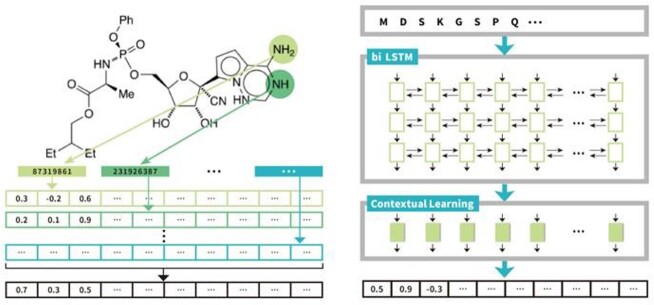
Principle of NLP-inspired deep learning-based methods to embed drug SMILES strings (Mol2vec) and protein sequences (ProSE). Similar drugs or proteins will be in close distance after the embedding because these methods can capture contextual information.

**Fig 2.**
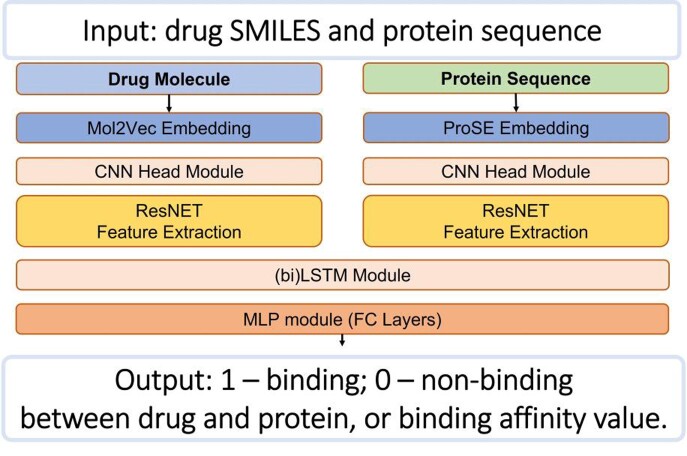
Model architecture from input to output of DeepLPI.

**Results:**

Results showed that the DeepLPI reached an overall AUROC of 0.79 based on the BindingDB internal test, 76% better than DeepCDA. DeepLPI also outperformed DeepCDA using the external Davis dataset (AUROC=0.53) and COVID-19 3CL Protease dataset (AUROC=0.61).

**Figure 3.**
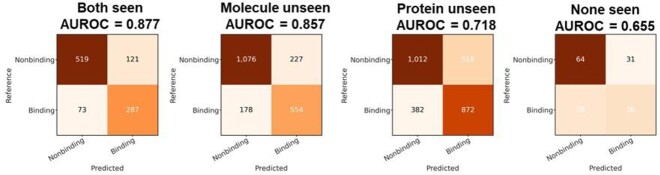
A randomly reserved part of the BindingDB dataset was grouped into four cases for testing, according to whether the drug molecules or the proteins are seen in the training set. (Note that the drug-target pairs in the test set are excluded from the training set.)

**Fig 4.**
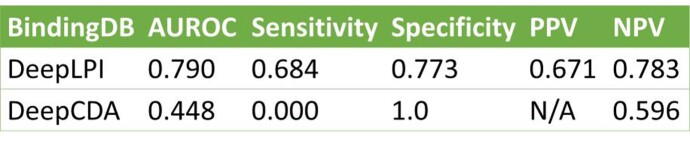
We compare with DeepCDA, a recently published DTI prediction model that reported to be better than previous models. DeepLPI is 76% better in accuracy metric AUROC in classification tasks.

**Fig 5.**
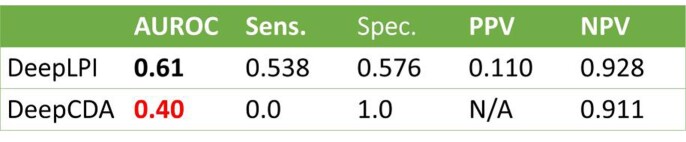
As a preliminary attempt, the model trained on BindingDB was applied to a recent COVID-19 dataset targeting the 3CL-protease, which reported 897 small molecule drugs. Our model is better than the baseline method by 25%.

**Conclusion:**

The high performance of DeepLPI suggests that our model has the potential to identify new COVID-19 drugs when applied to approved drugs. The generalizability of the model also promises applications to diseases in a wider scope.

**Disclosures:**

**All Authors**: No reported disclosures.

